# Lactate and lactylation modifications in neurological disorders

**DOI:** 10.4103/NRR.NRR-D-24-01344

**Published:** 2025-06-19

**Authors:** Yu Gu, Keyang Chen, Chunyan Lei, Xinglong Yang, Lu Wang, Linhu Zhao, Wen Jiang, Qionghua Deng

**Affiliations:** First Department of Neurology, First Affiliated Hospital of Kunming Medical University, Kunming, Yunnan Province, China

**Keywords:** astrocyte-neuron lactate shuttle theory, brain functions, brain lactate metabolism, central nervous system, histone lysine lactylation, monocarboxylate transporters, nervous system, neurodegenerative diseases, non-histone lysine lactylation, post-translational modifications

## Abstract

Research into lactylation modifications across various target organs in both health and disease has gained significant attention. Many essential life processes and the onset of diseases are not only related to protein abundance but are also primarily regulated by various post-translational protein modifications. Lactate, once considered merely a byproduct of anaerobic metabolism, has emerged as a crucial energy substrate and signaling molecule involved in both physiological and pathological processes within the nervous system. Furthermore, recent studies have emphasized the significant role of lactate in numerous neurological diseases, including Alzheimer’s disease, Parkinson’s disease, acute cerebral ischemic stroke, multiple sclerosis, Huntington’s disease, and myasthenia gravis. The purpose of this review is to synthesize the current research on lactate and lactylation modifications in neurological diseases, aiming to clarify their mechanisms of action and identify potential therapeutic targets. As such, this work provides an overview of the metabolic regulatory roles of lactate in various disorders, emphasizing its involvement in the regulation of brain function. Additionally, the specific mechanisms of brain lactate metabolism are discussed, suggesting the unique roles of lactate in modulating brain function. As a critical aspect of lactate function, lactylation modifications, including both histone and non-histone lactylation, are explored, with an emphasis on recent advancements in identifying the key regulatory enzymes of such modifications, such as lactylation writers and erasers. The effects and specific mechanisms of abnormal lactate metabolism in diverse neurological diseases are summarized, revealing that lactate acts as a signaling molecule in the regulation of brain functions and that abnormal lactate metabolism is implicated in the progression of various neurological disorders. Future research should focus on further elucidating the molecular mechanisms underlying lactate and lactylation modifications and exploring their potential as therapeutic targets for neurological diseases.

## Introduction

Lactate was traditionally considered a metabolic waste product generated under anaerobic conditions (Ferguson et al., 2018), perpetuating the stereotype that lactate formation lacks a biological function (Ippolito et al., 2019). The primary pathway for lactate production is glycolysis, whereby pyruvate is converted to lactate by lactate dehydrogenase under anaerobic conditions. This process typically occurs in skeletal muscles but can also take place in the brain, intestines, and red blood cells. Under abnormal physiological conditions, lactate production can also occur in the lungs, visceral organs, and white blood cells (Reddy et al., 2015). Elevated lactate levels can result from various diseases, including shock, sepsis, cardiac arrest, trauma, seizures, diabetic ketoacidosis, malignant tumors, and liver dysfunction (Andersen et al., 2013). Pellerin and Magistretti (1994) first proposed the astrocyte-neuron lactate shuttle (ANLS) theory, highlighting the active role of astrocytes in neuronal energy consumption and suggesting that glucose is primarily absorbed by astrocytes and metabolized into lactate through the glycolytic pathway (Chuquet et al., 2010). Furthermore, in the early 21^st^ century, Mintun et al. (2004) discovered that lactate may also support neuronal activity. Indeed, a recent study showed that lactate acts as a signaling molecule and, thus, plays a crucial role in the regulation of brain function (Scavuzzo et al., 2020). Specifically, in the central nervous system (CNS), lactate influences brain function through specific receptors (Proia et al., 2016). Brooks (2018) further expanded on the ANLS concept, establishing that lactate serves not only as a significant source of aerobic energy metabolism and gluconeogenesis but also as a key mediator in intercellular signaling. Through the ANLS mechanism, lactate plays a pivotal role in the interaction between neurons and astrocytes, highlighting its multifaceted role in the CNS.

Lactylation, a novel form of post-translational modification (PTM) first reported by Professor Yingming Zhao’s research group in 2019, is an important process in relation to lactate and its functions. This study revealed that lactate can act as a substrate for the PTMs of histones. Specifically, PTMs of histones are covalent modifications that occur after protein translation. These modifications can regulate various aspects of protein function, including protein activity, localization, folding, and interactions with other biomolecules, such as proteins, nucleic acids, and lipids (Han et al., 2018). Importantly, increasing research in this area has revealed that many critical life processes and the onset of diseases are not solely related to protein abundance but are also significantly regulated by various protein PTMs.

Notably, histone lysine lactylation (Kla) has been identified as a significant PTM, suggesting that lactate may serve as a promising therapeutic target for various human diseases (Wang et al., 2023c). Indeed, as an endogenous substance, lactate triggers gene expression in pro-inflammatory macrophages during bacterial infections to promote cellular homeostasis, thereby offering a new perspective on the regulation of immune homeostasis (Zhang et al., 2019). Subsequent research has further confirmed that lactylation plays a role in various biological processes, including tumor proliferation (Chaudagar et al., 2023), heart failure (Lazzeri et al., 2015; Uyar et al., 2020), autoimmune diseases (Caslin et al., 2021; Huang et al., 2024), neural excitation (Hagihara et al., 2021), neuroinflammation (Boland et al., 2018; Aldana, 2019), and neurodegenerative diseases (Le Douce et al., 2020; Schirinzi et al., 2020).

Neural regeneration is a crucial process for the repair and recovery of the nervous system, particularly following injury or in the context of neurological diseases, and recent research has highlighted the important roles of both lactate and lactylation in this regenerative process. Within the CNS, lactate acts as a vital energy source, especially when neural tissue is under stress. Following an injury or during repair, lactate levels in the microenvironment rise, and both neurons and glial cells can take up lactate and convert it to pyruvate through the action of lactate dehydrogenase B (LDHB). This conversion allows pyruvate to enter the tricarboxylic acid (TCA) cycle, where it is further metabolized to produce ATP, the energy currency of the cell. This energy is crucial for neuronal survival and axonal regeneration, thereby facilitating neural repair (Monsorno et al., 2023). Additionally, lactate functions as a signaling molecule, enabling communication between neural cells and their surrounding environment, including glial and immune cells (Vaccari-Cardoso et al., 2022). In the event of neurological disease or injury, immune cells infiltrate the affected area and produce lactate, and this lactate then influences the metabolic and functional states of the neural cells. Overall, this intercellular lactate signaling establishes a microenvironment conducive to neural regeneration. Furthermore, lactate may modulate the polarization and function of immune cells, maintaining them in a mildly inflammatory state that supports repair mechanisms and, thus, indirectly promoting neural regeneration (Caslin et al., 2021). A recent finding indicates that after spinal cord injury, there is an increase in both lactate levels and lactylation in the spinal cord, and this upregulation enhances microglial proliferation, scar formation, axonal regeneration, and motor recovery. These studies have highlighted the significant role of lactate in the repair processes following neural injury, as lactate not only serves as an energy source but also as a facilitator of axonal regeneration (Hu et al., 2024b). Moreover, in 2024, Professor Marco Simoes-Costa’s research group made a pivotal discovery regarding the effect of lactylation on neural regeneration by altering key proteins involved in this process. They found that lactylation is integral in activating developmental gene regulatory networks, thus contributing to the formation of the epigenomic landscape required for the expression of genes that are essential for the development of neural crest cells (Merkuri et al., 2024). Notably, this finding opens new avenues for understanding how lactylation can be used to enhance neural regeneration and develop therapeutic strategies for neurological conditions.

Overall, the objective of this work is to conduct a comprehensive review of the roles of lactate and lactylation modifications in various neurological systems, thereby offering a novel perspective on the mechanisms of neural regeneration. This review primarily focuses on the physiological and pathological processes associated with lactate and lactylation in neurological diseases, aiming to provide novel insights and strategies for the treatment of these conditions.

## Search Strategy

In this narrative review, a search was conducted on PubMed for articles published from the inception of the database until 2024 using the following keywords: lactate, lactylation modification, lactate metabolism, monocarboxylate transporters, astrocyte-neuron shuttle theory, brain function, histone modification, non-histone modification, Parkinson’s disease (PD), cerebral ischemic stroke, Alzheimer’s disease (AD), acute ischemic stroke (AIS), multiple sclerosis (MS), Huntington’s disease (HD), myasthenia gravis (MG), epilepsy (EP), and neonatal hypoxic-ischemic encephalopathy (HIE). The search results were manually screened based on the titles and abstracts. Articles related to the metabolic regulatory roles of lactate and lactylation modifications in neurological diseases were selected based on the aforementioned keywords. These articles focus on brain lactate metabolism, the specific roles of lactate in regulating brain function, protein lactylation modifications, and their relationships with various neurological diseases. Ultimately, 216 papers were included in the final analysis, comprising original research articles, review papers, and other relevant publications.

## Advances in Understanding the Metabolic Regulatory Role of Lactate

### Lactate isomers

Research on the role of lactate in physiology and medicine spans nearly a century. There are three commonly produced isomers of lactate: D-lactate, L-lactate, and DL-lactate (**Table 1**). DL-lactate is a racemic mixture of L-lactate and D-lactate, containing equal amounts of both isomers. In the human body, lactate primarily exists as L-lactate and D-lactate, which are enantiomers/stereoisomers. Due to the asymmetry of the carbon atom in humans, L-lactate is the predominant form (Li et al., 2022b), while D-lactate is present at micromolar concentrations, representing approximately 1% of the concentration of L-lactate (Levitt and Levitt, 2020).

**Table 1:** Three lactate isomers

	L-lactate	D-lactate	DL-lactate
Introduction to isomers	It is the predominant form of lactate found in the human body (Li et al., 2022b).	In the human body, the concentration of this substance is at the micromolar level, which is approximately 1% of the concentration of L-lactate (Levitt and Levitt, 2020).	Racemic mixture of L-lactate and D-lactate
Formation pathways	L-lactate is produced via the glycolytic pathway from carbohydrates and amino acids, particularly when there is a higher demand for oxygen and adenosine triphosphate than the cell can supply. This is commonly observed during intense exercise (Harmer et al., 2008) and infections (Levy et al., 2005).	It originates from the metabolism of carbohydrates and lipids (Brandt et al., 1984) and is also synthesized by intestinal bacteria (Levitt and Levitt, 2020).	Undescribed
Physiological functions	It has the potential to promote protein synthesis, enhance synaptic remodeling, and increase axonal excitability, while also contributing to improved memory.	Undescribed	Undescribed
Associated diseases and effects	Undescribed	Short Bowel Syndrome, Crohn’s disease, acute appendicitis, and bacterial infection	Various neurological symptoms have been reported, including altered mental status (confusion) (Soler Palacín et al., 2006; Gigante et al., 2012), dysarthria (Dahhak et al., 2008), ataxia (Munakata et al., 2010; Burski et al., 2013), slurred speech (Grünert et al., 2010; Gigante et al., 2012), disorientation (Grünert et al., 2010), and even coma. In more severe cases, these symptoms can lead to D-lactate acidosis encephalopathy (Kang et al., 2006).

Specifically, L-lactate is generated through the glycolytic pathway by the breakdown of carbohydrates and amino acids, particularly when the demand for oxygen and ATP exceeds the cellular supply, such as during intense exercise (Harmer et al., 2008) and infections (Levy et al., 2005). Additionally, L-lactate serves as a crucial substrate in neuronal oxidative metabolism, promoting protein synthesis, enhancing synaptic remodeling, and increasing axonal excitability during learning and memory formation (Levitt and Levitt, 2020). L-lactate also contributes to memory enhancement (Suzuki et al., 2011). Conversely, D-lactate is derived from the metabolism of carbohydrates and lipids (Brandt et al., 1984) and is also produced by the gut bacteria (Levitt and Levitt, 2020). Excessive levels of D-lactate can lead to D-lactate acidosis under certain inflammatory conditions or infections, such as short bowel syndrome (Bianchetti et al., 2018; Khrais et al., 2022), Crohn’s disease (Cai et al., 2019), acute appendicitis (Filiz et al., 2010), and bacterial infections (Marcos et al., 1991). The CNS is also affected by D-lactate, and thus, excessive D-lactate levels can result in various neurological symptoms (Angelet et al., 2002; Kowlgi and Chhabra, 2015), including altered mental status (confusion) (Soler Palacín et al., 2006; Gigante et al., 2012), dysarthria (Thurn et al., 1985), ataxia (Munakata et al., 2010; Burski et al., 2013), slurred speech (Grünert et al., 2010; Gigante et al., 2012; Khrais et al., 2022), disorientation (Grünert et al., 2010), and even coma. In more severe cases, an excess of D-lactate can lead to D-lactate encephalopathy, which can cause neurotoxicity in the cerebellum. This condition can also result in decreased attention, hallucinations, and weakness (Kang et al., 2006).

### Metabolic regulatory role of lactate in disease progression

In 2011, Blad’s team discovered a family of G-protein-coupled receptors (GPCRs) known as hydroxycarboxylic acid receptors, which include hydroxycarboxylic acid receptor 1 (HCA1), hydroxycarboxylic acid receptor 2 (HCA2), and hydroxycarboxylic acid receptor 3 (HCA3). These receptors are collectively referred to as the niacin receptor family and constitute a cluster of GPCRs with high sequence homology (Blad et al., 2011). Among them, HCA1 is widely recognized as a key lactate sensor in adipose tissue and other peripheral organs (Liu et al., 2009; Brown and Ganapathy, 2020). This receptor functions by downregulating the formation of cyclic adenosine monophosphate (cAMP), thereby inhibiting lipolysis. This mechanism not only reduces the release of lipolytic products but also promotes the storage of energy-rich metabolites in adipocytes (Ahmed et al., 2009, 2010).

Subsequently, in 2014, Lauritzen’s team reported strong staining of G protein-coupled receptor 81 (GPR81) throughout the brain using sagittal slices from adult male wild-type C57BL/6 mice. The authors found that GPR81 was prominently expressed in Purkinje neurons and their dendrites in the cerebellum. This study confirmed the presence of the GPR81 receptor in the brain, and showed that this receptor can be activated by physiological concentrations of lactate and the specific GPR81 agonist, 3,5-dihydroxybenzoic acid, to reduce cAMP levels. Furthermore, both L-lactate and D-lactate can regulate neural network activity by binding to the GPR81 receptor (Bozzo et al., 2013; Wagner et al., 2015). By mediating the effects of L-lactate, GPR81 plays a crucial role in various physiological and pathological processes, including by promoting wound healing (Porporato et al., 2012), enhancing angiogenesis (Porporato et al., 2012), providing neuroprotection (Schurr et al., 2001; Shen et al., 2015), supporting cancer cell survival in the tumor microenvironment (Roland et al., 2014), reducing inflammatory responses (Hoque et al., 2014; Madaan et al., 2017), promoting immunomodulation (Hoque et al., 2014), and inhibiting lipolysis (Liu et al., 2009).

Lactate exhibits bidirectional functions in neurons and glial cells, particularly astrocytes, under varying conditions. Lactate can have potentially harmful effects on the CNS. For example, studies have shown that increased lactate uptake by neurons promotes the production of reactive oxygen species (ROS), enhances mitochondrial energy metabolism, and leads to oxidative stress in neurons. This oxidative stress not only negatively affects ATP synthesis but also further increases ROS production in the mitochondria, creating a negative cycle that ultimately results in axonal degeneration in the peripheral nervous system (Jia et al., 2021). Conversely, lactate is essential for maintaining brain homeostasis. Indeed, research has demonstrated that HCA1 is highly expressed in the CNS and has neuroprotective functions. For example, lactate reduces neuroexcitotoxic damage by activating HCA1-mediated signaling, suggesting that it plays a significant role in regulating overall brain metabolism (Laroche et al., 2021).

In terms of neurological disorders, in 2019, Lu et al. reported that lactate levels in the hippocampus and cerebral cortex were significantly reduced in a mouse model of AD. This finding was in line with the results of a 2018 study, which showed that injecting lactate into the hippocampal region in day-old chicks could alleviate the memory retention deficits caused by neural damage induced by 1,4-dideoxy-1,4-imino-D-arabinitol (Alberini et al., 2018). The brain-derived neurotrophic factor (*BDNF*) gene, associated with cognitive improvement and the alleviation of depressive symptoms, plays a crucial role in these processes (El Hayek et al., 2019). Lactate has also been shown to work as a neuroprotective factor (Bezzi and Volterra, 2011; Newman et al., 2011) by activating the silent information regulator 1-dependent induction of the peroxisome proliferator-activated receptor-gamma coactivator-1 alpha (PGC-1α)/fibronectin type III domain-containing protein 5 pathway. This activation enhances BDNF signaling in the hippocampus, promoting learning and memory formation. Furthermore, in 2019, El Hayek’s research team discovered that exercise increased the levels of endogenous lactate crossing the blood–brain barrier (BBB). Finally, Li et al. (2020a) reported that L-lactate plays a crucial role in neural repair and functional recovery after spinal cord injury, as the local application of L-lactate to the injured spinal cord was found to significantly promote corticospinal tract axon regeneration and accelerate behavioral recovery in adult mice.

Multiple studies have demonstrated that lactate plays a multifaceted role in metabolic reprogramming (Rabinowitz and Enerbäck, 2020; Wang et al., 2023c) and epigenetic modifications (Peixoto et al., 2020). Lactate does not simply serve as an energy source for mitochondrial respiration; indeed, a research team led by Zhang et al. in 2019 discovered a novel modification method via mass spectrometry in human and mouse cells: the lactylation of histone H3 at lysine 18 (H3K18la). Histone lactylation represents a novel lactate-mediated PTM pathway. Specifically, histones undergo lactylation in the cell nucleus, where lactate is transferred to the lysine residues on proteins with the assistance of acetyltransferases. This process results in the formation of lactylation groups that alter the structure and activity of histones (Zhang et al., 2019; Moreno-Yruela et al., 2022).

## Brain Lactate Metabolism

Brain cells primarily consist of neurons and glial cells. Neurons are essential cells within the CNS and are responsible for receiving and transmitting electrochemical signals through neurotransmission. While neurons perform essential functions, approximately 90% of the cells in the CNS are glial cells, which include astrocytes, oligodendrocytes, ependymal cells, and microglia (Allen and Lyons, 2018). Among these, astrocytes outnumber neurons in the human brain (Freeman, 2010) and play crucial roles in various physiological functions. Both neurons and glial cells are involved in highly specialized metabolic processes. Despite being an organ with high energy consumption, the brain has very limited energy reserves and relies heavily on a continuous supply of energy substrates (Peters, 2011). Any disruption in this energy supply can lead to neurological dysfunction, loss of consciousness, and even coma within minutes.

In particular, glucose is an essential nutrient for the adult brain and is delivered via the bloodstream and transported across the BBB into cells through sodium-dependent glucose cotransporters (SGLT1 and SGLT2) and sodium-independent glucose transporters (GLUT1 and GLUT3) (Leiz et al., 2013; Mergenthaler et al., 2013). Once inside the cells, glucose is phosphorylated by hexokinase to form glucose-6-phosphate, which is subsequently processed through various metabolic pathways. Since the demand for energy within the brain, particularly in neurons, is extremely high, a continuous supply of glucose is required to maintain normal function (Mergenthaler et al., 2013). Although the human brain constitutes about 2% of the total body weight, it consumes approximately 25% of the body’s glucose-derived energy (Castro et al., 2009), utilizing about 5.6 mg of glucose per 100 g of brain tissue per minute. Therefore, the brain is highly dependent on glucose, meaning glucose homeostasis is crucial for its normal physiological functions.

By generating ATP, glucose metabolism provides energy for the brain’s physiological activities and is, thus, important for maintaining the basic functions of the neuronal and non-neuronal cells and synthesizing neurotransmitters. The primary metabolic pathways involved in glucose metabolism in the brain include: (1) glycolysis (the most common pathway), which leads to the production of lactate or further metabolism of pyruvate in the mitochondria; (2) the pentose phosphate pathway (PPP), which provides nucleotides and the reduced form of nicotinamide adenine dinucleotide phosphate (NADPH); and (3) gluconeogenesis, which primarily occurs in astrocytes. Specifically, astrocytes exhibit significant 6-phosphofructo-2-kinase/fructose-2,6-bisphosphatase-3 activity, a key regulatory mechanism for glycolysis and gluconeogenesis. Together, these pathways collectively ensure that the brain has a continuous supply of energy to support its high metabolic demands and various physiological functions.

**Figure 1:**
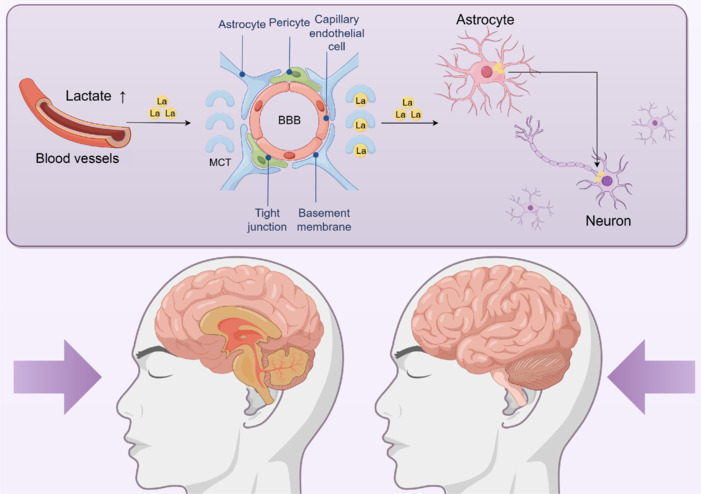
Lactate transport. When lactate levels in the blood rise, the brain becomes a net lactate uptake organ. MCTs on the BBB facilitate the transport of lactate from the blood into the brain. According to the astrocyte-neuron lactate shuttle theory, astrocytes and oligodendrocytes jointly extract glucose from the blood and mobilize glycogen, releasing lactate under the command of neurons. BBB: Blood–brain barrier; La: lactate; MCT: monocarboxylate transporter.

Discoveries made in the early 20^th^ century by Warburg and his research team revealed the existence of the Warburg effect, whereby reprogramming of glucose metabolism in cancer cells increases the rate of lactate production increases and acidifies the tumor microenvironment, thus creating favorable conditions for the rapid proliferation and growth of tumor cells (Hanahan and Weinberg, 2011). Even in the presence of sufficient oxygen, tumor cells and many other types of proliferating cells prefer to utilize glycolysis for energy, thus generating large amounts of lactate. This discovery fundamentally overturned the notion of lactate as merely a metabolic waste product (Dienel, 2012). When at rest, the brain acts as a lactate sensor, and when blood lactate levels rise, the brain demonstrates net lactate uptake. Monocarboxylate transporters (MCTs) on the BBB facilitate the transport of lactate from the blood into the brain. According to the ANLS hypothesis (Suzuki et al., 2011), astrocytes and oligodendrocytes work together to extract glucose from the blood and mobilize glycogen, thus causing the release of lactate under the direction of the neurons (**Figure 1**). Neurons maintain active pyruvate dehydrogenase, which channels pyruvate into the Krebs cycle. In contrast, pyruvate dehydrogenase in astrocytes is inhibited by phosphorylation, diverting pyruvate toward lactate production and release (Halim et al., 2010).

**Figure 2:**
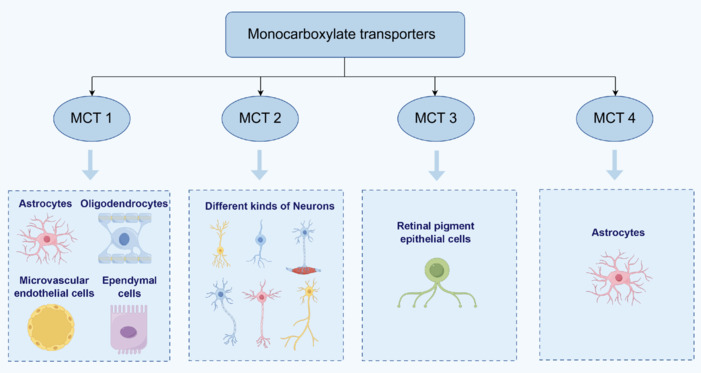
Distribution of MCTs. The MCT family is widely distributed across organisms and is primarily responsible for the transmembrane transport of monocarboxylates such as lactate, pyruvate, and ketone bodies. These compounds are metabolic products generated within the body and are crucial components of energy metabolism. MCT1 is mainly expressed in astrocytes, microvascular endothelial cells, ependymal cells, and oligodendrocytes. MCT2 is primarily located in neurons, while MCT3 is predominantly found in retinal pigment epithelial cells. MCT4 is almost exclusively expressed by astrocytes. MCT: Monocarboxylate transporter.

MCTs are a class of membrane carriers specialized in transporting monocarboxylates, such as lactate, to meet the energy demands of neurons during activity. MCT1 is primarily expressed in astrocytes, microvascular endothelial cells, ependymal cells, and oligodendrocytes (Simpson et al., 2007; Rinholm et al., 2011; Domènech-Estévez et al., 2015). In contrast, MCT2 is predominantly localized in neurons, while MCT4 is almost exclusively expressed by astrocytes (Pierre and Pellerin, 2005; **Figure 2**). Under physiological conditions, the coordinated action of MCTs 1–4 facilitates the shuttling of lactate between glycolytic and oxidative cells, which is crucial for maintaining lactate homeostasis within different tissues (Li et al., 2022b). During neuronal activity, glutamate is released into the synaptic cleft, prompting the release of glucose from blood vessels. This glucose is then absorbed by astrocytes, which convert the glucose into pyruvate through glycolysis. Pyruvate is subsequently converted into lactate by lactate dehydrogenase isoenzyme A (LDHA), and the lactate is shuttled to neurons via MCTs 1 and 4, where it serves as a precursor for gluconeogenesis. This lactate transfer mechanism, explained by the ANLS hypothesis (Lee et al., 2022), provides the necessary energy for neurons and is crucial for maintaining neuronal metabolism and neurotransmitter transmission (Suzuki et al., 2011). Regarding disease processes, astrocytes may play dual roles in various neurological disorders by potentially promoting or inhibiting inflammation and neurodegeneration (Giovannoni and Quintana, 2020). For example, astrocytes demonstrate active functions and are considered key regulatory factors in diseases such as MS (Itoh et al., 2018; Linnerbauer et al., 2020), AD (Wheeler et al., 2019; Preman et al., 2021) and PD (Ramos-Gonzalez et al., 2021; Chen et al., 2023; Wang et al., 2023b).

Under specific conditions, such as during certain developmental stages or under starvation, the brain can utilize alternative energy substrates from the blood, including ketone bodies (Nehlig, 2004). Additionally, during intense exercise, the brain can secrete lactate as an energy source (van Hall et al., 2009). Indeed, although glucose is recognized as the primary energy source for the brain, circulating lactate serves as a supplementary source. When blood glucose levels are insufficient, lactate can fulfill the energy demands of an active brain (Dienel, 2019). Schurr et al. (1988) reported that lactate could support synaptic transmission in brain slices in the absence of glucose. More recently, Lhomme et al. (2021) reported that lactate can directly support neuronal activity, as they discovered that when the lactate shuttle in hypothalamic ependymal-glial cells was inhibited, the energy balance of pro-opiomelanocortin neurons was disrupted. Consequently, lactate, rather than glucose, is necessary to maintain the activity of pro-opiomelanocortin neurons.

In fact, every function of the brain relies on the support or regulation of lactate. Research has highlighted specific instances of the brain’s reliance on lactate that cover a wide range of phenomena and processes, from the cellular level to the organ level. At the cellular level, lactate plays a crucial role in the myelination process of oligodendrocytes (Rinholm et al., 2011; Fünfschilling et al., 2012; Lee et al., 2012) and serves as an important regulator of calcium signaling within astrocytes (Requardt et al., 2012). At the organ level, lactate is involved in the physiological regulation of ventilation (Erlichman et al., 2008) and plays a significant role in memory formation (Newman et al., 2011; Suzuki et al., 2011). Overall, these findings highlight that lactate is indispensable to various aspects of brain metabolism and function.

## Specific Roles of Lactate in Regulating Brain Function

Lactate plays various roles in the regulation of brain function, serving as an energy substrate, a signaling molecule, and a modulator of epigenetic modifications. These roles are essential for normal brain function and influence the progression of neurological diseases (**Table 2**).

**Table 2:** Specific roles of lactate in regulating brain function

Classification of actions	Relevant studies and findings
Influences the processes of learning and memory	Lactate regulates synaptic plasticity and activity-dependent neuronal gene expression (Suzuki et al., 2011).
	Astrocytes regulate lactate supply to support neuronal function and modulate memory formation (Newman et al., 2011; Gold et al., 2013).
	L-lactate enhances NDMA receptor currents, modulates the neuronal redox state, and increases the expression of plasticity-related genes (Yang et al., 2014).
	The physiological injection of L-lactate increases BDNF expression and enhances learning and memory in mice (El Hayek et al., 2019).
	D-lactate impairs memory by inhibiting the uptake of L-lactate or disrupting pyruvate metabolism (Baker and Edwards, 2007; Gibbs and Hertz, 2008).
	HCAR1 agonists or D-lactate post-training enhance memory, while pre-training D-lactate impairs and L-lactate enhances memory (Scavuzzo et al., 2020).
	Post-training administration of HCAR1 agonists or D-lactate enhances memory, while pre-training administration of D-lactate impairs memory (Scavuzzo et al., 2020).
Mediates mitochondrial metabolism in the brain	Lactate enhances the expression of MCT1 and cytochrome c oxidase, increases the activity of transcription factor activity, and upregulates genes associated with oxidative stress and mitochondrial metabolism (Hashimoto et al., 2007).
	Neural precursor cells in the developing neocortex produce and secrete lactate via anaerobic glycolysis to regulate angiogenesis and cell division (Dong et al., 2022).
	High concentrations of lactate can promote mitochondrial fusion and inhibit mitochondrial fission (Hu et al., 2021).
	Lactate enters mitochondrial matrix, stimulates electron transport chain activity, and inhibits glycolysis (Cai et al., 2023).
	Lactate can enhance mitochondrial autophagy in non-small cell lung cancer cells (Wang et al., 2023a).
Affects neuronal activity	Activation of GPR81 regulates PLC activity and inhibits L-lactate-induced neuronal excitability (de Castro Abrantes et al., 2019).
	Neuronal excitation can induce protein lactylation in mouse brain cells (Hagihara et al., 2021).
	Excessive lactate intake by neurons induces mitochondrial dysfunction, oxidative stress, and peripheral axonal degeneration (Yang et al., 2021).
Neuroprotective effects	L-lactate upregulates the HCA1 receptor in the cortex and striatum and provides neuroprotection alongside D-lactate under ischemic conditions (Castillo et al., 2015).
	L-lactate promotes the unfolded protein response, activates NRF2, and protects cells from oxidative stress (Tauffenberger et al., 2019).
	Lactate transporters mediate neuroprotection through neuronal activation, with both exogenous and endogenous lactate demonstrating protective effects (Cerina et al., 2024).

BDNF: Brain-derived neurotrophic factor; GPR81: G protein-coupled receptor 81; HCA1/HCAR1: hydroxycarboxylic acid receptor 1; MCT1: monocarboxylate transporter 1; NDMA: N-methyl-D-aspartate receptor; NRF2: nuclear factor-erythroid 2-related factor 2; PLC: phospholipase C.

### Influence of lactate on learning and memory

Suzuki et al. (2011) reported that lactate is important in the formation of long-term memory in mice, likely due to its regulation of synaptic plasticity and activity-dependent neuronal genes. Newman et al. (2011) assessed brain glucose and lactate levels in Sprague-Dawley rats using the spontaneous alternation test and reported that during spatial working memory tasks, extracellular glucose levels decreased, while lactate levels increased. Furthermore, injecting lactate into the hippocampus enhanced the rats’ memory performance, and inhibiting glycogen phosphorylase with 1,4-dideoxy-1,4-imino-D-arabinitol to limit lactate production in astrocytes impaired memory, although this impairment could be reversed by lactate supplementation. Notably, blocking lactate transport to neurons via MCT2 using α-cyano-4-hydroxycinnamate also impaired memory, but this impairment could not be reversed by lactate supplementation. Taken together, these findings confirm that astrocytes support neuronal function by regulating lactate supply, thereby modulating memory formation. Gold et al. (2013) provided further support for this concept by demonstrating that lactate provision in astrocytes is not only critical for memory consolidation but also plays a broader role in the processes of learning and memory. In the following year, another study revealed that L-lactate enhances N-methyl-D-aspartic acid A-mediated currents, leading to an increase in intracellular calcium. Concurrently, L-lactate elevated intracellular levels of nicotinamide adenine dinucleotide, thereby modulating the redox state of neurons. These results suggest that L-lactate increases the expression of plasticity-related genes in neurons, such as activity-regulated cytoskeleton-associated protein, zinc finger binding protein clone 268, fos proto-oncogene, and BDNF, by regulating N-methyl-D-aspartic acid receptor activity, which is associated with changes in the cellular redox state (Yang et al., 2014). Further research by El Hayek et al. (2019) demonstrated in mice that intraperitoneal injections of physiological concentrations of L-lactate (117 or 180 mg/kg) for 1 month can increase the protein and gene expression of BDNF, thereby improving learning and memory abilities.

Additionally, as early as the beginning of the 21st century, studies using newly hatched chicks demonstrated that D-lactate impairs memory through two pathways: by inhibiting the uptake of L-lactate into astrocytes (i.e., an extracellular effect) and by disrupting the metabolism of pyruvate in astrocyte mitochondria (i.e., an intracellular effect) (Baker and Edwards, 2007; Gibbs and Hertz, 2008). Subsequently, Scavuzzo et al. (2020) reported that lactate plays different roles at various stages of memory. Specifically, using an inhibitory avoidance memory protocol in rats, the authors found that a post-training subcutaneous injection of the specific hydroxycarboxylic acid receptor 1 (HCAR1) agonist 3,5-dihydroxybenzoic acid (3,5-DHBA) or D-lactate significantly enhanced memory, whereas L-lactate had no effect. Conversely, D-lactate administered 15 minutes before training impaired memory, while those rats treated with L-lactate 15 minutes prior to training exhibited improved memory.

### Lactate mediation of mitochondrial metabolism in the brain

In 2007, Hashimoto et al. first identified in L6 cells that lactate can increase the messenger ribonucleic acid and the protein expression of both MCT1 and cytochrome c oxidase. Lactate also enhances the deoxyribonucleic acid binding activity of the transcription factors nuclear factor-erythroid 2-related factor 2 and cAMP response element binding protein, both of which are involved in mitochondrial gene expression. Importantly, the authors also found that 20 mM lactate significantly upregulated genes related to the oxidative stress response, including glutathione peroxidase, MCT1, and CD147. Additionally, lactate-sensitive genes involved in mitochondrial metabolism were found to be upregulated, including the genes for ATP synthase (ATP5g1 and ATP5o), NADH dehydrogenase, translocase of the inner membrane, the succinate dehydrogenase complex, hydroxyacyl-CoA dehydrogenase, and the cytochrome c oxidase subunit 4I1 (Hashimoto et al., 2007). Subsequently, other research teams have reported that during early development, neural precursor cells in the mouse neocortex, specifically radial glial precursor cells, synthesize and secrete large amounts of lactate through anaerobic glycolysis. In the developing mouse neocortex, lactate stimulates vascular growth and simultaneously regulates mitochondrial morphology, collectively modulating vascular development and the division behavior of radial glial precursor cells (Dong et al., 2022).

Additionally, L-lactate-mediated mitochondrial metabolism has also been observed in primary mouse neurons. For example, in 2021, Hu et al. investigated the role of lactate in promoting mitochondrial function during high-intensity interval training using primary cultured mouse hippocampal cells. Their study found that treatment with 15 mM L-lactate for 3 hours significantly increased ATP levels and the expression of oxidative phosphorylation-related genes in these cells. Notable upregulated genes included those for ubiquinol-cytochrome c reductase core protein 1, ATP synthase F1 subunit alpha, ubiquinone oxidoreductase core subunit S8, succinate dehydrogenase, and cytochrome c oxidase subunit 5B. The expression of BDNF protein was also increased in mouse hippocampal cells. Furthermore, the study revealed that the expression of mitochondrial fusion proteins 1 and 2 was significantly upregulated, while the expression of dynamin-related protein 1 and mitochondrial fission 1 protein was inhibited. However, there were no significant changes in the expression of mitophagy-related proteins, such as PINK1, Parkin RING-between-RING E3 ubiquitin-protein ligase, and sequestosome 1, indicating that high concentrations of lactate can promote mitochondrial fusion and inhibit mitochondrial fission with minimal impact on mitophagy (Hu et al., 2021).

In 2023, Cai et al. reported that lactate can enter the mitochondrial matrix and stimulate the activity of the mitochondrial electron transport chain. Indeed, in their work, both L-lactate and D-lactate were found to effectively enhance electron transport chain activity and inhibit glycolysis, confirming that lactate is a key regulatory factor in mitochondrial oxidative phosphorylation that suppresses the fermentative capacity of glucose. Wang et al. (2023a) demonstrated that the mitochondrial copy number in both normoxic and hypoxic non-small cell lung cancer cells (A549) decreased as the lactate concentration increased, whereas the mitochondrial copy number in human normal lung epithelial cells (BEAS-2B) exhibited the opposite trend. This finding suggests that lactate may enhance the mitophagy function in non-small cell lung cancer cells. These findings are significant for advancing the current understanding of brain energy metabolism and mitochondrial mechanisms, and future research should further explore these areas at both the animal model and cellular levels.

### Effect of lactate on neuronal activity

A study published in 2019 investigated the intracellular pathways involved in HCAR1 activation using primary cortical neurons from wild-type (WT) and HCAR1 knockout (KO) mice (de Castro Abrantes et al., 2019). The findings revealed that GPR81 in neurons plays a crucial role in regulating L-lactate-induced spontaneous neuronal activity. Specifically, the activation of GPR81 causes it to bind to the Giβ subunit, which then regulates the activity of phospholipase C. This interaction induces hyperpolarization through potassium ion (K^+^) influx or activates γ-aminobutyric acid (GABA) receptors, thereby reducing neuronal excitability (de Castro Abrantes et al., 2019). A subsequent study confirmed that neuronal excitation can induce lactylation modifications of proteins in the mouse brain. Specifically, lysine lactylation in brain cells was found to be co-regulated by lactate levels, neuronal excitation, and behavior-related variables. Overall, Hagihara et al. (2021) identified 63 lysine-lactylated proteins, noting that stress preferentially increased the lactylation of histone H1.

A recent study indicated that prolonged excessive lactate intake by neurons can lead to mitochondrial metabolic disorders in peripheral glial cells, specifically Schwann cells, resulting in the increased production of ROS. Consequently, this oxidative stress in the neurons affects mitochondrial function and ATP synthesis, ultimately causing peripheral axon degeneration. This study highlights the dual effects of lactate on neuronal activity; indeed, while moderate lactate levels can provide energy support to neurons, excessive lactate can compromise the integrity of peripheral axons (Yang et al., 2021). Given these findings, the connection between lactate and neuronal activity is becoming increasingly clear. In fact, numerous research teams have conducted extensive studies on the mechanisms and specific pathways by which lactate influences various neurological disorders (see the section entitled “Lactate, Lactylation Modifications, and Neurological Disorders” for details).

### Neuroprotective effects of lactate

When investigating the mechanisms underpinning the role of lactate in neurological disorders, researchers commonly utilize various models, including the brain ischemia-reperfusion model (middle cerebral artery occlusion, MCAO) (Castillo et al., 2015; Yao et al., 2023), the traumatic brain injury (TBI) model (Zhai et al., 2020), and chronic neurodegenerative disease models such as AD (Zhang et al., 2018; Sun et al., 2020; Pan et al., 2022) and PD (Li et al., 2022a). Additionally, other frequently used models (**Table 3**) include neonatal rat hypoxic-ischemic encephalopathy models (Roumes et al., 2021) and *in vitro* hypoxia and ischemia models, such as the oxygen-glucose deprivation model (Berthet et al., 2009).

**Table 3:** Research on lactate and lactylation modifications in various nervous system disease models

Models	Animals/Cells	Conclusions
MCAO model	ICR-CD1 mice (Berthet et al., 2009)	Immediate lactate injection reduces striatal lesion size and improves neurological outcomes post-reperfusion.
	12-wk-old male C57/BL6 mice (Hu et al., 2012)	High lactylation promotes pro-inflammatory to anti-inflammatory microglial shift, enhancing neurotrophic factors, neuroplasticity, and neurogenesis.
	Male CD1 mice with tFCI induced by MCAO (Castillo et al., 2015)	L-lactate injection during reperfusion enhances HCA1 receptor expression and neuroprotection in the cortex and striatum.
OGD model	P12 rat (Berthet et al., 2009)	4 mM L-lactate reduces neuronal death post-OGD, while 20 mM is toxic.
AD model	Three-mon-old heterozygous APP/PS1 mice (Zhang et al., 2018)	Reduced brain lactate and MCT expression hinder lactate transport from glia to neurons, worsening neuronal energy deficiency.
	5XFAD mice (Pan et al., 2022)	Increased brain lactate and histone lactylation, with elevated H4K12la in microglia near Aβ plaques.
PD model	MPTP-induced PD mouse models and MPP treated SH-SY5Y cells (Li et al., 2022a)	The upregulation of HK2 and LDHA, along with elevated lactate levels, promotes the apoptosis of dopaminergic neurons.
HIE model	Rice-Vannucci mature hypoxic-ischemic model P7 pup (Roumes et al., 2021)	Lactate injection reduces brain lesion volume by 30%, demonstrating rapid neuroprotection; however, this effect is abolished by oxamate, indicating that it depends on metabolic utilization rather than signaling.
TBI model	Rat (Zhai et al., 2020)	L-lactate preconditioning significantly increases the expression of GPR81, PSD95, GAP43, BDNF, and MCT2 in the cortex and hippocampus 24 hours after TBI, alleviating the neurological deficits induced by TBI in rats.
CIRI model	Rat (Yao et al., 2023)	In the context of CIRI, key proteins SLC25A4 (K245) and SLC25A5 (K96) within the Ca^2+^ signaling pathway undergo lactylation, whereas the lactylation of VDAC1 (K20, K266) is eliminated.
IS model	Human induced pluripotent stem cells (Cerina et al., 2024)	Astrocyte activation and endogenous lactate production are associated with neuroprotection; however, this effect is diminished when lactate transporters are inhibited. Additionally, the supplementation of L-lactate prior to hypoxic conditions significantly improves cell viability.
HD model	STHdhQ7 and STHdhQ111 cell lines (Solís-Maldonado et al., 2018)	Ascorbic acid stimulates the uptake of lactate by inhibiting glucose transport, and the overexpression of GLUT3 restores this effect in HD cells. This suggests that alterations in GLUT3 are responsible for the inefficient utilization of lactate in HD neurons.

5XFAD: 5-Mutation familial Alzheimer’s disease; AD: Alzheimer’s disease; APP/PS1: amyloid precursor protein/presenilin-1; Aβ: amyloid-beta; BDNF: brain-derived neurotrophic factor; CIRI: cerebral ischemia-reperfusion injury; GAP43: growth-associated protein 43; GLUT3: glucose transporte 3; GPR81: G protein-coupled receptor 81; H4K12la: histone 4 lysine 12 lysine acetylation; HCA1: hydroxycarboxylic acid receptor 1; HD: Huntington's disease; HIE: hypoxic-ischemic encephalopathy; HK2: hexokinase 2; IS: ischemic stroke; LDHA: lactate dehydrogenase A; MCAO: middle cerebral artery occlusion; MCT: monocarboxylate transporter; MCT2: monocarboxylate transporter 2; MPP: 1-methyl-4-phenylpyridinium; MPTP: 1-methyl-4-phenyl-1,2,3,6-tetrahydropyridine; OGD: oxygen-glucose deprivation; PD: Parkinson’s disease; PSD95: postsynaptic density protein 95; SLC25A4: solute carrier family 25 member 4; SLC25A5: solute carrier family 25 member 5; STHdhQ111: striatal tissue with 111 glutamine repeats of Huntington’s disease protein; STHdhQ7: striatal tissue with 7 glutamine repeats of Huntington’s disease protein; TBI: traumatic brain injury; tFCI: transient focal cerebral ischemia; VDAC1: voltage-dependent anion channel 1.

Both *in vivo* and *in vitro* models have shown that L-lactate exhibits neuroprotective effects. For instance, Castillo et al. (2015) demonstrated that an intravenous injection of L-lactate in an MCAO model enhanced the expression of HCA1 receptors in the cortex and striatum. Both L-lactate and D-lactate have neuroprotective effects under ischemic conditions, a function that involves HCAR1 activation, while 3,5-DHBA can reduce cell death. In 2019, Tauffenberger et al. utilized neuroblastoma cells (SH-SY5Y) as an *in vitro* model and found that L-lactate promotes the unfolded protein response and activates nuclear factor erythroid 2-related factor 2. By inducing a mild burst of ROS, L-lactate triggers pathways that are related to impaired glucose tolerance, protein kinase B expression, phosphoinositide 3-kinase activation, and endoplasmic reticulum stress, thereby protecting cells from oxidative stress *in vitro* (Tauffenberger et al., 2019). Subsequently, another research team established an *in vitro* cell model of the human ischemic penumbra and identified that lactate transporters are involved in the neuroprotective effects mediated by neuronal activation (Cerina et al., 2024). In particular, the authors found that the exogenous administration of lactate before hypoxia was neuroprotective, as was stimulating astrocytes to produce endogenous lactate. Notably, these findings provide a potential theoretical basis for the development of treatments for ischemic stroke.

## Protein Lactylation Modifications

**Figure 3:**
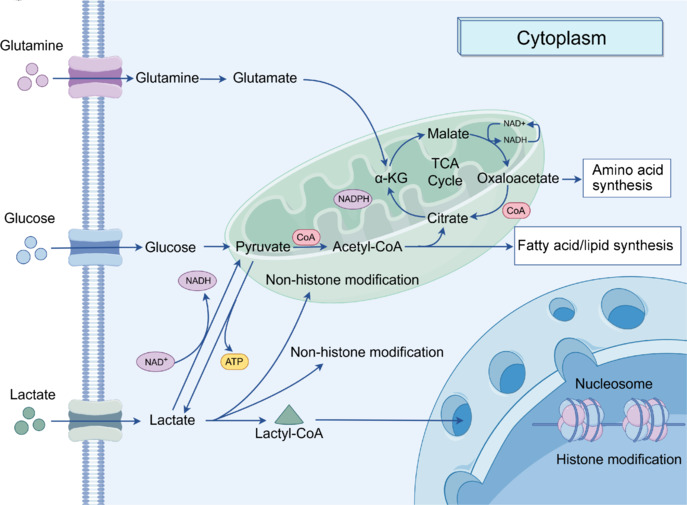
Pathways of lactate in maintaining neuronal metabolic regulation. Lactate can be catabolized via two pathways. First, lactate is oxidized to pyruvate, which then enters the mitochondria and is metabolized through the TCA cycle. This process involves the irreversible conversion of lactate to pyruvate by the enzyme pyruvate dehydrogenase. Second, lactate can be converted into glucose through gluconeogenesis. Additionally, lactate can be transformed into lactyl-CoA, which participates in the lactylation of histones within nucleosomes and non-histone proteins located in the cytoplasm and mitochondria. α-KG: Alpha-ketoglutarate; ATP: adenosine triphosphate; CoA: coenzyme A; NAD^+^: nicotinamide adenine dinucleotide; NADH: reduced nicotinamide adenine dinucleotide: TCA: tricarboxylic acid.

Neuronal activity throughout the brain requires substantial metabolic resources (Yellen, 2018). Lactate is produced via glycolysis or the breakdown of glutamine and is transported into astrocytes through MCTs. Within cells, lactate is catabolized through two pathways. First, lactate is oxidized to pyruvate, which then enters the mitochondria and is metabolized via the TCA cycle; second, under hypoxic conditions, lactate is generated through gluconeogenesis. Additionally, lactate can be converted into lactyl-CoA and can then participate in the lactylation of histones and non-histone proteins (Zhu et al., 2024; **Figure 3**).

### Histone modification of regulatory proteins

**Figure 4:**
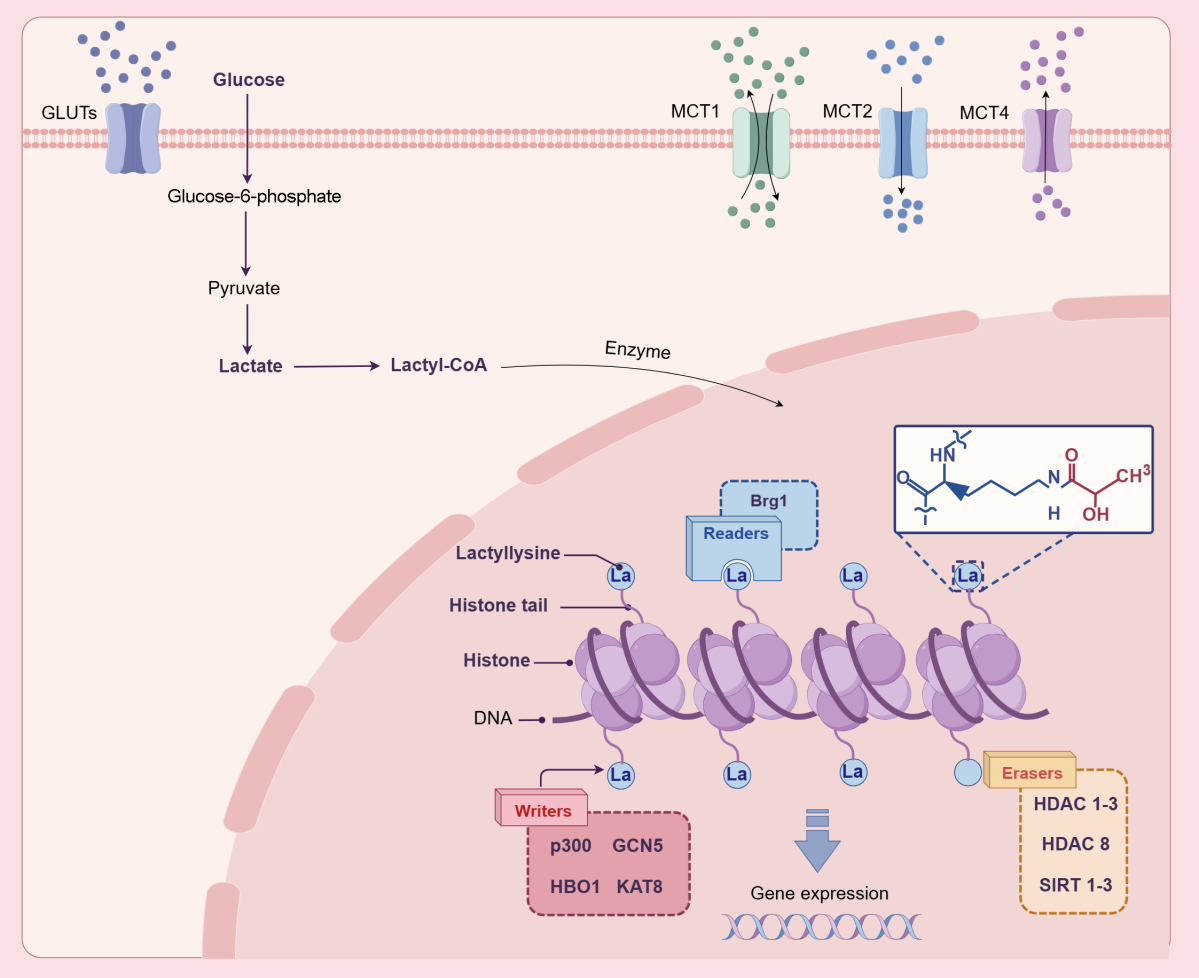
Regulatory proteins involved in histone modification. The regulation of histone modifications can be classified into three categories of proteins: Writers, Erasers, and Readers. Lactylation writers are enzymes that catalyze the addition of lactyl groups to proteins. These enzymes include p300, GCN5, HBO1, and KAT8. Lactylation readers are proteins that recognize and bind to lactylated modifications, thereby regulating gene expression and other cellular processes; the only lactylation reader reported to date is Brg1. Lactylation erasers are enzymes that remove lactyl groups from proteins, including HDAC1–3, HDAC8, and SIRT1–3. Collectively, these writers, readers, and erasers participate in the dynamic regulation of lactylation modifications, influencing a variety of biological processes, including gene expression, protein function, and cellular metabolism. Brg1: Brahma-related gene 1; DNA: deoxyribonucleic acid; GCN5: general control nonderepressible 5; GLUTs: glucose transporters; HBO1: histone acetyltransferase binding to ORC1; HDAC1: histone deacetylase 1; HDAC2: histone deacetylase 2; HDAC3: histone deacetylase 3; HDAC8: histone deacetylase 8; KAT: lysine acetyltransferase; La: lactate; MCT: monocarboxylate transporter; SIRT: sirtuin.

In recent years, the field of research on lactylation has made significant advancements, providing new insights into the role of lactylation in both physiological and pathological processes. PTMs regulate protein function and can be controlled through enzymatic or non-enzymatic mechanisms, with enzymatic regulation being the most common. Moreover, the proteins that regulate histone modifications can be classified into three categories: writers, erasers, and readers (**Figure 4**).

Several studies have focused on the critical regulatory enzymes involved in lactylation during enzymatic reactions and have successfully identified numerous lactylation writers and erasers respectively, including certain acetyltransferases and deacetylases. Emerging research suggests that the lysine acetyltransferase (KAT) family can catalyze various acylations, including those by p300/CREB-binding protein (CBP) and Gcn5-related N-acetyltransferase (GNAT). In 2015, Dancy and Cole reported that protein lysine acetylation can be accomplished through the action of p300/CBP. Subsequently, in 2019, Zhang et al. made the initial discovery of p53-dependent p300-mediated lactylation of histones H3 and H4 in macrophages. This finding highlighted that p300, a well-known acetyltransferase, can facilitate both acetylation and lactylation. L-lactate can be converted into L-lactoyl-CoA (Varner et al., 2020), which is subsequently transferred to histones by acetyltransferases such as p300 (Zhang et al., 2019). The p300/CBP complex, as a lactylation writer, plays a significant role in regulating histone lactylation in macrophages and in the induction of pluripotent stem cells (Li et al., 2020b). Another study suggested that general control non-repressible 5 (GCN5), a member of the GNAT family, catalyzes histone modifications. Furthermore, the transport of lactate facilitated by MCT1 promotes histone lactylation, thus contributing to tissue repair and the recovery of cardiac function following myocardial infarction (Wang et al., 2022). Another lactylation writer, histone acetyltransferase binding to origin recognition complex subunit 1 (HBO1), functions as a lactyltransferase that can regulate histone lactylation both *in vitro* and within cellular environments. Specifically, HBO1 mediates histone lysine lactylation-dependent gene transcription. Further analyses utilizing CUT&Tag technology showed that histone H3K9la lactylation at transcription start sites requires HBO1, which preferentially catalyzes the lactylation of histone H3K9l (Niu et al., 2024).

Histone deacetylases 1–3 (HDAC1–3) have demonstrated robust activity against L- and D-lactyl lysine (K(L-la) and K(D-la)), as well as various short-chain acyl modifications. For example, in a laboratory study, Moreno-Yruela et al. (2022) illustrated that HDAC3 was the most efficient enzyme for removing both L- and D-lactyl lysine. Additionally, Jennings et al. (2021) experimentally showed that Sirtuin 2 (SIRT2) functions as an enzymatic deacylase for lactyl lysine modifications. This finding highlights the critical role of SIRT2 in the PTM of lactyl lysine, thus demonstrating the effectiveness of SIRT2 as an eraser for this modification. Furthermore, their study provides new insights into the enzymatic regulatory mechanisms underpinning non-enzymatically derived protein PTMs (Jennings et al., 2021). Finally, Fan et al. (2023) observed that Sirtuin 3 (SIRT3) exhibited enhanced eraser activity at the histone H4 lysine 16 lactylation (H4K16la) site compared to other sirtuins.

In 2024, Hu et al. (2024a) first reported that the Brahma-related gene 1 (Brg1) protein functions as a reader of histone lactylation. Through proteomic analysis of immunoprecipitation experiments targeting histone H3 lysine 18 lactylation (H3K18la), Hu et al. (2024a) demonstrated the specific recruitment of Brg1 during cellular reprogramming. Notably, in their work, both H3K18la and Brg1 exhibited significant enrichment at the promoter regions of genes associated with pluripotency and epithelial cell junctions. This finding highlights the synergistic role of H3K18la and Brg1 in regulating gene expression, thereby laying the groundwork for further investigation into histone lactylation readers and providing new insights into the molecular mechanisms underpinning cellular reprogramming.

Simultaneously, the newly discovered enzyme responsible for catalyzing the synthesis of lactoyl-CoA has garnered significant attention in the scientific community. Research has identified that alanyl-tRNA synthetase (AARS) also functions as an enzyme facilitating the process of histone lactylation. AARS1, which acts as a lactate sensor, mediates global lysine lactylation in tumor cells, and this lactylation can modify p53, promote tumorigenesis, and is associated with poor prognosis in cancer patients (Zong et al., 2024). In the same year, Ju et al. (2014) reported that AARS1 functions as a genuine lactyltransferase, directly utilizing lactate and ATP to facilitate protein lactylation. Furthermore, Li et al. (2014) reported in cellular and mouse models that under L-lactate stimulation, AARS2 binds to cyclic guanosine monophosphate-adenosine monophosphate synthase, mediating its lactylation and inactivation. This finding confirms that AARS1 and AARS2 act as intracellular L-lactate sensors and play crucial roles as lactyltransferases. The study elucidated a two-step reaction mechanism underpinning the AARS-catalyzed lactylation modification, which involves the formation of a lactate-AMP intermediate through an ATP-dependent reaction, followed by the transfer of lactate to the lysine residues of target proteins. Additionally, Zhu et al. (2024) demonstrated that ACSS2 functions as a lactate coenzyme A synthetase, facilitating the conversion of lactate into acetyl-CoA. This process involves the formation of a functional complex with lysine acetyltransferase 2A, which is involved in the development of glioblastoma. Specifically, this complex significantly enhances cellular proliferation and immune evasion while accelerating the growth of brain tumors.

The identification of these enzymes offers a novel direction for future research into the mechanisms of lactylation, including in relation to neurodegenerative diseases. In this context, this research advancement has the potential to significantly enhance precision medicine.

### Histone lactylation modification

In 2019, Zhang et al. identified a novel epigenetic modification known as Kla. This modification is generated by aerobic glycolysis following the polarization of pro-inflammatory macrophages. When specific enzymes are active, L-lactyl-CoA, an activated form of L-lactate, serves as a substrate for histone lactylation (K(L-a)). The lactylation of 28 lysine residues on histones has been shown to regulate gene expression. In particular, studies have confirmed that histone Kla accumulates on gene promoters and, thus, promotes lactate production when cells are subjected to stimuli such as hypoxia, interferon-gamma, lipopolysaccharides, or bacterial attack (Irizarry-Caro et al., 2020; Tyl et al., 2025). Overall, this modification plays a direct and significant role in the regulation of gene expression (Allis and Jenuwein, 2016).

Another research team confirmed that histone lysine crotonylation (Kcr) and Kla are widely distributed throughout the brain and undergo significant changes during neurodevelopment; additionally, these dynamic changes are closely linked to the regulation of neuronal function and epigenetic modifications (Dai et al., 2022). In 2022, Pan et al. made a breakthrough in potential therapeutic approaches for AD by discovering that the level of histone H4 lysine 12 lactylation (H4K12la) was significantly elevated in microglia located adjacent to amyloid-beta (Aβ) plaques in mice. This lactate-dependent histone modification rapidly accumulated at the promoters of glycolytic genes, thus activating transcription and increasing glycolytic activity. Ultimately, this process created a positive feedback loop involving glycolysis, H4K12la, and pyruvate kinase M2, which then exacerbated microglial dysfunction in AD. Based on these findings, the pharmacological inhibition of pyruvate kinase M2 could be used to reduce microglial activation, disrupt the positive feedback loop, and enhance spatial learning and memory functions in mice with AD. In the future, further in-depth research on this drug should be conducted, with the goal of applying it to human patients with Alzheimer’s disease.

However, research on the epigenetic regulation of genes through histone lactylation modification in the nervous system is still in its early stages, and further in-depth exploration of this topic is required in the future.

### Non-histone lactylation modification

In 2020, Gaffney et al. identified 350 lactoylated proteins in Human Embryonic Kidney 293T (HEK293T) cells using liquid chromatography-tandem mass spectrometry. Their findings showed that under non-enzymatic conditions, lactoyl-glutathione was transferred to lysine residues on proteins, typically resulting in K(D-la) modifications on non-histone proteins. Two research teams have also reported that the acetyl group from acetyl-CoA can be transferred to the ε-amino group of lysine through a non-enzymatic, pH-driven acylation mechanism (Wagner and Payne, 2013; James et al., 2017). This acyl transfer mechanism is considered a primary contributor to mitochondrial lysine acetylation.

In 2021, Hagihara et al. identified 63 lysine-lactylated proteins in the mouse brain and reported that brain lactate levels are regulated by systemic changes in brain cells, neural excitation, and behavior-related stimuli. These behavior-related stimuli include several experimental manipulations: intraperitoneal lactate injection, electroconvulsive treatment, and exposure to a novel, anxiety-provoking open environment. The research team used cell type-specific markers for the dual immunostaining of Kla and identified Kla immunoreactivity in dopaminergic neurons, astrocytes, and microglia. This finding suggests that Kla may be widespread among neurons, thus providing evidence for protein lactylation in the brain and its regulation by lactate levels, which themselves are influenced by neural activity (Hagihara et al., 2021). In 2022, Wan et al. (2022) identified cyclic ammonium ions of lactoyl-lysine during tandem mass spectrometry analysis, further revealing extensive lactylation in the human proteome. Furthermore, a recent study indicated that TBI could induce the lactylation modification of the Tufm protein at the K286 site, which then hindered its binding with the mitochondrial transport protein Tomm40, and this inhibition of mitophagy resulted in increased neuronal apoptosis (Weng et al., 2024).

In summary, these studies on protein lactylation modifications have demonstrated that extensive lactylation occurs in the proteome beyond histones and plays a significant role in regulating protein functions. Importantly, further comprehensive investigations into lactylation modification sites and mechanisms in non-histone proteins may provide novel targets for the treatment of neurological disorders.

## Lactate, Lactylation Modifications, and Neurological Disorders

**Figure 5:**
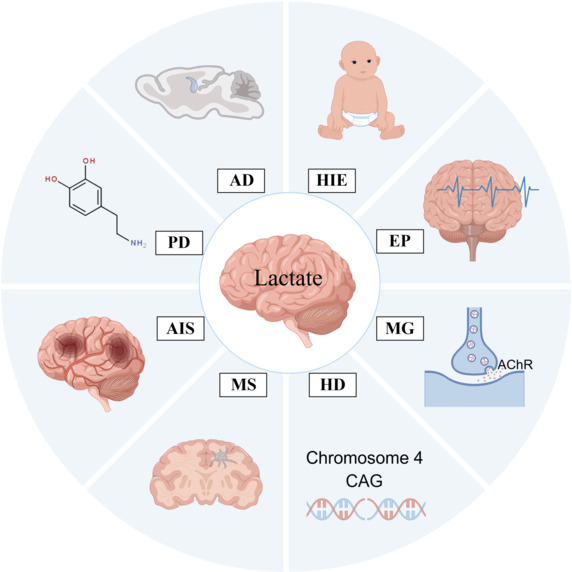
Association between lactate, lactylation modifications, and nervous system diseases. Abnormal lactate metabolism may be implicated in neurological disorders such as AD, PD, AIS, MS, HD, MG, EP and HIE. AChR: Acetylcholine receptor; AD: Alzheimer’s disease; AIS: acute ischemic stroke; CAG: cytosine-adenine-guanine; EP: epilepsy; HD: Huntington’s disease; HIE: hypoxic-ischemic encephalopathy; MG: myasthenia gravis; MS: multiple sclerosis; PD: Parkinson’s disease.

Lactylation modifications are gaining increasing attention in the medical field. Indeed, a recent study highlighted that lactate functions as a signaling molecule in the regulation of brain functions (Scavuzzo et al., 2020), and this finding has opened new directions for the treatment of various neurological conditions. Numerous studies have indicated that abnormal lactate metabolism may be implicated in several neurological disorders, including AD, PD, AIS, MS, HD, MG, EP, and HIE in neonates (**Figure 5**).

### Alzheimer’s disease

AD, a prevalent and debilitating neurodegenerative disease among the aging population (Erkkinen et al., 2018), is the most common form of dementia, accounting for 60%–80% of all dementia cases (Revi, 2020). The primary characteristics of AD include progressive cognitive impairment and dementia (Brookmeyer et al., 2018). The median survival time from the onset of dementia ranges from 3.3 years to 11.7 years (Todd et al., 2013). Pathologically, AD is characterized by both the deposition of Aβ plaques and tau neurofibrillary tangles in the brain (Ferreiro et al., 2006; Huang and Mucke, 2012; Ferrari and Sorbi, 2021). In particular, the accumulation of Aβ plaques is a key pathological feature of AD and leads to both neuronal loss and damage to axons and synapses (Knopman et al., 2021). The “amyloid hypothesis” posits that the deposition of Aβ in the brain is the primary driver of AD progression and is associated with declines in behavioral and cognitive functions (Selkoe and Hardy, 2016).

In 2010, based on findings from a mouse model with mitochondrial DNA (mtDNA) mutations, Ross et al. demonstrated that chronically elevated lactate levels are characteristic of aging and dementia. Additionally, lactate accumulation is closely associated with the apoptosis of cerebellar granule cells in the early stages of AD (Bobba et al., 2015). Two other studies have revealed that lactate levels are significantly elevated in the cerebrospinal fluid (CSF) of patients with AD and mild cognitive impairment, with these findings suggesting that the dysregulation of glucose metabolism may play a critical role in the onset and progression of AD (Liguori et al., 2015; Xiang et al., 2021).

Native lactate dehydrogenase (LDH) is composed of four subunits of either LDHA or LDHB, forming different LDH isoenzymes (Markert, 1963). Notably, the LDHA isoenzyme functions in anaerobic glycolysis by catalyzing the conversion of pyruvate to lactate, while the LDHB isoenzyme primarily catalyzes the conversion of lactate to pyruvate (Li et al., 2022b). Harris et al. (2016) reported that an increased intracellular LDHA/LDHB ratio promotes lactate accumulation within cells. The enhancement of the glycolytic pathway and the subsequent increase in lactate production may represent a protective response of the brain to Aβ toxicity. As previously stated, astrocytes provide energy to neurons in the form of lactate, and the presence of MCT4 in astrocytes and MCT2 in neurons is essential for the transfer of lactate between these cells (Figley, 2011). Deficits in the lactate shuttling process from glial cells to neurons could lead to metabolic dysregulation in the brain, resulting in neurodegenerative changes similar to those observed in AD (Sun et al., 2020). In 2018, Parkin et al. introduced the concept of the ANLS, with glutamate transporters being central to this transport mechanism.

Glutamate is the most common excitatory neurotransmitter in the CNS (Parkin et al., 2018). A study conducted by Erin and colleagues revealed that the presence of soluble Aβ_42_ leads to an increase in the release of glutamate, and concurrently, the activation of the ANLS enhances the rate at which glutamate is cleared, resulting in higher levels of extracellular lactate. This research provides insight into the interaction between glutamate and lactate, emphasizing that glutamate released by neurons is removed from the extracellular space through excitatory amino acid transporters located on glial cells. The uptake of glutamate is linked to the transport of glucose into astrocytes, which subsequently enhances glycolysis. The lactate produced is then transported from astrocytes to neurons via MCTs, effectively integrating glutamatergic neurotransmission with bioenergetics to support synaptic signaling (Hascup et al., 2022).

Additionally, in 2019, Wang et al. confirmed in mice that brain endothelial cells play a crucial role in maintaining lactate homeostasis and regulating adult hippocampal neurogenesis. The authors found that increased lactate accumulation impaired hippocampal neurogenesis and showed that the phosphatase and tensin homolog/protein kinase B pathway in endothelial cells promotes lactate transport across the brain endothelium by enhancing the expression of MCT1. Furthermore, overexpression of MCT1 in the brain vasculature or the loss of protein kinase B1 can restore MCT1 expression in phosphatase and tensin homolog mutant mice, reduce lactate levels, and normalize hippocampal neurogenesis and cognitive function.

In summary, studying changes in lactate metabolism may offer a new strategy for the development of interventions targeting AD (Zhao and Xu, 2022). However, further research is needed to explore the evidence for how lactate could be targeted in the clinical treatment of patients with AD.

### Parkinson’s disease

PD is a chronic, progressive neurodegenerative disorder and the second most common neurodegenerative disease after AD. PD is characterized by the degeneration of dopaminergic neurons and the presence of Lewy bodies in the substantia nigra pars compacta. The loss of these dopaminergic neurons results in decreased dopamine levels in the brain, which adversely affects motor control and coordination and leads to a range of motor dysfunctions, including tremors and muscle stiffness (McGregor and Nelson, 2019; Rees et al., 2019). PD is also associated with non-motor symptoms, such as cognitive decline (Armstrong and Okun, 2020).

A clinical study conducted in 2022 indicated that patients with PD (*n* = 101) exhibited significantly elevated lactate levels in their cerebrospinal fluid (CSF) compared to a control group (*n* = 60). This increase in lactate was correlated with clinical disease progression and neurodegenerative biomarkers, such as tau protein and dopamine, along with the dopamine metabolite 3,4-dihydroxyphenylacetic acid (Liguori et al., 2022). In recent years, CSF lactate levels have garnered significant attention in PD research (Nakano et al., 2017), and these levels are currently considered a potential indicator for assessing neuronal metabolic pathways in both early-onset and late-onset forms of the disease (Schirinzi et al., 2020). Indeed, various studies have suggested that lactate levels in the CSF may serve as reliable indicators of brain metabolic damage.

In 2020, Schirinzi et al. discovered that CSF lactate levels were abnormally elevated in patients with late-onset PD. The following year, Li et al. (2022a) conducted *in vivo* and *in vitro* experiments using a neurotoxin model induced by 1-methyl-4-phenyl-1,2,3,6-tetrahydropyridine in mice, as well as the human neuroblastoma cell line SH-SY5Y, and found that the upregulation of hexokinase 2 and LDHA led to increased lactate levels, thus promoting the apoptosis of dopaminergic neurons. Conversely, inhibiting hexokinase 2 expression can reduce lactate production in PD and regulate the adenosine monophosphate-activated protein kinase/protein kinase B/mammalian target of rapamycin signaling pathway, thereby reducing the apoptosis of dysfunctional neurons (Li et al., 2022a). Glycolysis converts glucose into pyruvate, which then enters the Krebs cycle in the mitochondria (Liguori et al., 2016; Bonomi et al., 2021), and impairments in this metabolic process can result in abnormal brain energy metabolism, leading to elevated CSF lactate levels. Additionally, this elevation in CSF lactate levels is associated with a decrease in CSF dopamine levels. Overall, as PD progresses, the concentration of lactate in the CSF may reflect the degree of neurodegeneration, and thus, lactate could potentially serve as a biomarker for future clinical or experimental research.

In summary, these studies have indicated that lactate plays a crucial role in the apoptosis of dopaminergic neurons in PD, and reducing lactate production may potentially alleviate the progression of the disease.

### Acute ischemic stroke

Stroke is a major cause of permanent disability and death worldwide (Benjamin et al., 2017), and approximately 87% of strokes are ischemic in nature (Liao et al., 2020). In an ischemic stroke, the interruption of the brain’s blood supply leads to hypoxia and nutrient deprivation, triggering a series of pathophysiological changes. Although reperfusion can restore the oxygen and nutrient supply, this also induces oxidative stress, inflammatory responses, and apoptosis, which further exacerbate brain tissue damage. Therefore, ischemia/reperfusion (I/R) injuries are a key cause of the neuronal damage associated with AIS.

Glycolysis plays a crucial role in neuronal metabolism during ischemic stroke, as during such an event, lactate becomes an important energy source for neurons (Schurr et al., 1997). Research has demonstrated that endogenously produced lactate during brain ischemia has significant neuroprotective effects (Schurr et al., 2001), and increasing evidence suggests that lactate is essential for neuronal recovery following ischemia. In 2009, an international research team reported that administering 4 mM of L-lactate to rat hippocampal slices subjected to oxygen-glucose deprivation significantly reduced neuronal death; however, a higher dose of 20 mM L-lactate was found to be toxic to neurons (Berthet et al., 2009). Additionally, in an *in vivo* mouse model of MCAO, a lactate injection into the contralateral ventricle immediately after reperfusion significantly reduced the lesion size in the striatum and improved neurological outcomes. Overall, this study confirmed that the early administration of low-dose lactate post-ischemia can prevent neuronal death, reduce lesion size, and enhance neurological function (Castillo et al., 2015).

In glial cells, the excessive production of lactate can cause intracellular lactate accumulation, thus triggering a series of biological responses, including the activation of inflammatory reactions, increased oxidative stress, the initiation of apoptosis, and excitotoxicity (Chen et al., 2011). These processes may collectively lead to further damage and dysfunction of the nervous system. Indeed, a study has shown that in a mouse model of transient MCAO (tMCAO), the intravenous administration of lactate during the reperfusion period of brain ischemia provides neuroprotection and enhances the expression of HCA1 receptors in the cerebral cortex and striatum (Castillo et al., 2015). MCT1 facilitates the transport of lactate from astrocytes and oligodendrocytes to neurons, thus promoting the entry of lactate into the TCA cycle (Rose et al., 2020). In 2019, Xu et al. determined that the upregulation of MCT1 during ischemic stroke plays a protective role in I/R injuries. Indeed, enhanced glycolysis leads to excessive lactate production, and MCT1 may help balance lactate distribution in ischemic brain cells by regulating lactate transport. Therefore, the metabolic function of MCT1 supports neuronal survival, and moreover, MCT1 may mitigate risk factors associated with I/R injury by reducing the intracellular accumulation of excess lactate. Conversely, if the MCT1 receptors are dysfunctional and cannot effectively regulate lactate transport, this may activate harmful cellular responses under I/R injury conditions. Consistent with a 2022 study, under ischemic conditions, MCT1 may alleviate glial cell damage caused by lactate accumulation by promoting the transport of lactate from glial cells to neurons (Zhang et al., 2022). Additionally, MCT1 can provide the necessary metabolic support to neurons to help them meet their energy demands. This process may play a crucial role in neuroprotection following brain ischemic injury.

Two recent studies have further explored the effect of lactate accumulation in the brain during ischemic periods and its influence on brain injury outcomes in AIS. In particular, Zhou et al. (2024) discovered that in astrocytes, low-density lipoprotein receptor-related protein 1 reduced the transfer of mitochondria to neurons and alleviated the impact of ischemic stroke by inhibiting the lactylation of adenosine diphosphate-ribosylation factor 1 (Zhou et al., 2024). Subsequently, Xiong et al. (2024) demonstrated that the pharmacological inhibition of lactate production, either by inhibiting LDHA or glycolysis, significantly reduced brain injury in mice with ischemic stroke. These authors found that the increase in brain lactate derived from astrocytes exacerbated ischemic brain injury by promoting the formation of lactylated proteins. This finding suggests that inhibiting lactate production or the formation of lactylated proteins during the ischemic phase may represent new therapeutic targets for treating ischemic stroke.

### Multiple sclerosis

MS is a chronic, inflammatory CNS disease characterized by inflammation and demyelination (Milo and Kahana, 2010) and is currently the most common non-traumatic disabling disease affecting young adults (Berger et al., 2017; Reich et al., 2018). Typical symptoms associated with MS include, but are not limited to, monocular vision loss due to optic neuritis, limb weakness or sensory loss caused by transverse myelitis, diplopia resulting from brainstem dysfunction, and ataxia due to cerebellar lesions (Brownlee et al., 2017). Between 2009 and 2014, various researchers demonstrated that mitochondrial dysfunction and impaired energy metabolism are considered significant contributors to axonal degeneration and the progression of MS (Trapp and Stys, 2009; Campbell and Mahad, 2011; Witte et al., 2014).

In 2016, Albanese et al. collected data from 118 subjects with relapsing-remitting MS to evaluate certain clinical indicators of MS disease severity and progression, including the progression index, MS severity score, and Bayesian risk estimate. The results indicated that CSF lactate levels were elevated in patients with MS and negatively correlated with disease severity. Furthermore, CSF lactate levels showed a significant positive correlation with CSF levels of tau protein and neurofilament light protein, suggesting a relationship between lactate levels and neurodegenerative processes in MS. Ghareghani et al. (2016) demonstrated that lactate levels in either CSF or serum could serve as a useful biomarker for reflecting treatment response and disease severity in MS patients with MS.

In summary, measuring CSF lactate levels, along with other biomarkers of tissue damage, could serve as a predictor of disease severity in patients with MS. Further research into related mitochondrial metabolic pathways may offer insights for new therapeutic targets.

### Huntington’s disease

HD is an autosomal dominant neurodegenerative disorder caused by the excessive expansion of cytosine-adenine-guanine repeat sequences in the huntingtin gene on chromosome 4 (The Huntington’s Disease Collaborative Research Group, 1993). This mutation leads to the aggregation of misfolded mutant huntingtin protein in the striatum and cortex, causing a range of symptoms, including dystonia, involuntary movements, dementia, cognitive impairment, personality changes, and behavioral issues. These symptoms contribute to a decline in health that ultimately results in death (Wyant et al., 2017).

As mentioned previously, MCTs play a central role in lactate uptake in the brain. Solís-Maldonado et al. (2018) conducted a study on the expression and function of MCTs in STHdhQ7 and STHdhQ111 cell lines to investigate potential impairments in neuronal lactate transport in HD. The study demonstrated that HD neurons can express the highly efficient MCT2, which facilitates increased lactate uptake during neuronal activity. However, the expression of the neuronal glucose transporter 3 (GLUT3) was found to be reduced in HD cell lines, resulting in inhibited glucose uptake. This inhibition can be attributed to the increased uptake of ascorbate induced by active synaptic neurons, which then suppresses glucose transport and leads to elevated glutamate release from glial cells (Castro et al., 2009).

Due to the ANLS mechanism, neurons primarily consume glucose as their energy source during resting states. However, during synaptic activity, neurons preferentially utilize lactate to meet increased energy demands. Glutamate released by neurons stimulates astrocytes to take up glucose, thereby enhancing glycolysis (Pellerin and Magistretti, 1994) and facilitating the release of lactate (Magistretti and Allaman, 2015). This process also leads to the depletion of ascorbic acid in astrocytes. Castro et al. (2009) demonstrated that ascorbic acid may act as a metabolic switch by inhibiting glucose consumption during glutamatergic synaptic activity. Ascorbic acid achieves this outcome by inhibiting glucose transport through a GLUT3-dependent mechanism and promoting lactate uptake in neurons (Castro et al., 2008). Alterations in energy metabolism in both the resting and active brain are characteristic features of the prodromal and early stages of HD (Felipe et al., 2012).

In HD, increasing the expression of GLUT3 and providing ascorbic acid supplementation may enhance the neuronal uptake of lactate, and thus, this strategy could potentially represent a new research direction for preventing the onset of HD and slowing its progression.

### Myasthenia gravis

MG is an autoimmune disease characterized by dysfunction at the neuromuscular junction, which causes the body’s immune system to attack acetylcholine receptors. The symptoms of MG include fluctuating muscle weakness that affects the ocular, bulbar, limb, and respiratory muscles and often worsens with activity and improves with rest. In 1975, patten first reported the presence of lactate in the blood of MG patients and suggested that lactate might be one of the causes of muscle weakness. Subsequently, Patten et al. (1974) investigated the underlying mechanisms of this phenomenon, revealing the existence of pathological changes linked to the binding of lactate with calcium ions in MG, which reduced the concentration of ionic calcium at the neuromuscular junction and the overall serum calcium levels. The decrease in serum calcium ions was found to impair the release of acetylcholine, thus severely affecting neuromuscular function. Brodsky and Smith (2007) reported that after foot surgery under spinal anesthesia in patients with MG, the release of lactate during the deflation of bilateral tourniquets exacerbated the condition, suggesting that lactate not only contributes to muscle weakness but also potentially worsens symptoms.

In recent years, there has been limited research on the potential link between lactate and MG. Therefore, future researchers in this field should explore the specific mechanisms involved in this relationship. Such investigations may offer new insights and directions for slowing the progression of MG.

### Epilepsy

Epilepsy is a chronic brain disorder characterized by recurrent seizures, which often lead to damage in neurons and glial cells (Betjemann and Lowenstein, 2015). This disorder is marked by excessive neuronal excitability and sudden synchronous discharges, resulting in recurrent, episodic, and transient dysfunctions of the CNS. Multiple studies have identified increased concentrations of L-lactate in various brain regions during epileptic seizures, including the gray and white matter (Thoresen et al., 1998), CSF (Mariani et al., 2020), cerebral cortex (Slais et al., 2008), and hippocampus (During et al., 1994). As early as 2005, an international research team reported that patients with medial temporal lobe epilepsy exhibited elevated extracellular lactate concentrations during the interictal period (Cavus et al., 2005). In 2017, Angamo et al. further investigated this phenomenon, demonstrating using rat hippocampal slices that endogenous lactate supports synaptic signaling and plays a crucial role in the temporal progression of the extracellular ion balance following neuronal activation. Their research also demonstrated a reduction in stimulus-induced changes in partial oxygen pressure (∆pO_2_) after the application of MCT inhibitors within both chronic epileptic tissue and rat hippocampal slices, indicating a significant role for lactate in oxidative metabolism.

In 2019, another research team investigated the effects of lactate on subicular pyramidal neurons in rat hippocampal slices using an *in vitro* epilepsy model. This team also examined the signaling mechanisms associated with the inhibition of epileptiform discharges by lactate. The study found that epileptic seizures were linked to elevated lactate concentrations, which increased from 2 mmol/L to 6 mmol/L in the hippocampus and cortex during seizures. Lactate was also shown to act as a neuroprotective agent by influencing subicular neurons through the hydroxycarboxylic acid receptor 1 and G protein-coupled inwardly rectifying potassium channels. Overall, this finding highlights the significant role of elevated brain L-lactate in epilepsy (Jorwal and Sikdar, 2019).

In 2021, Magnusson et al. evaluated the diagnostic value of prehospitalization lactate levels in 383 patients experiencing transient loss of consciousness. Their study showed that lactate levels exceeding 2.45 mmol/L, measured in the emergency department within 2 hours of the transient loss of consciousness, were indicative of epileptic seizures. Furthermore, the research demonstrated that the prehospitalization analysis of blood lactate levels was useful for identifying epileptic seizures in these cases, although this variable was found to lack specificity.

Currently, the specific pathways and mechanisms of the role of lactate in epilepsy remain under-researched. Based on previous clinical and experimental data, L-lactate receptor signaling may potentially represent a new target for antiepileptic therapy that warrants further investigation.

### Hypoxic-ischemic encephalopathy

Neonatal HIE is a severe neurological disorder that occurs during the neonatal period (Zhou et al., 2022) and is one of the most common causes of neonatal death and adverse neurodevelopmental outcomes globally (Blair and Watson, 2006). A study conducted in 2012 indicated that in neonatal HIE, ischemic neurons promote the polarization of microglia toward a pro-inflammatory phenotype, thus exacerbating the neuronal loss induced by oxygen-glucose deprivation. Conversely, maintaining microglia in an anti-inflammatory phenotype can protect neurons from oxygen-glucose deprivation through cell–cell contact, which is crucial in tissue repair, remodeling, and angiogenesis. Importantly, high levels of lactylation modification can induce the transformation of microglia from the pro-inflammatory type to the anti-inflammatory type, resulting in the production of various neurotrophic factors that promote neuroplasticity and neurogenesis (Hu et al., 2012).

In 2019, Zhang et al. confirmed that both endogenous and exogenous lactate contribute to lysine lactylation. Moreover, in 2020, Ivashkiv et al. reported that lactate can act within the nucleus through histone lactylation and inhibit signaling pathways under hypoxic conditions, thereby suppressing the activation of inflammatory macrophages and promoting the polarization of anti-inflammatory macrophages, which are important for maintaining homeostasis. In the same year, another research team demonstrated that lactate administration could reduce brain lesion volume, improve behavioral outcomes, and enhance long-term memory in rat models of neonatal hypoxia-ischemia (Tassinari et al., 2020). In 2021, Roumes et al. used the Rice-Vannucci model of mature hypoxia-ischemia in postnatal day 7 (P7) pups to study the neuroprotective effects of an intraperitoneal injection of lactate (40 μmol). The authors assessed the brain lesion size and apparent diffusion coefficient using magnetic resonance diffusion-weighted imaging and found that a single lactate injection reduced brain lesion volume by 30%, consistent with the findings of Tassinari’s team in 2020.

Therefore, it can be concluded that lactate administration may represent a novel and effective neuroprotective strategy for treating neonatal HIE.

## Limitations

Previous research in this field has predominantly focused on exploring the association between lactate, lactylation, and neurological disorders, particularly in relation to common neurological diseases such as AD and PD. For example, numerous researchers have conducted in-depth investigations into the potential impact of lactate and lactylation on key pathological processes in AD, including Aβ plaque deposition, tau protein phosphorylation, and neuronal apoptosis, with the aim of understanding the pathogenesis of AD and identifying novel therapeutic based on metabolism and PTMs. Moreover, in PD research, the focus has largely been on understanding the mechanisms by which lactate and lactylation influence the maintenance of dopaminergic neuron function, mitochondrial homeostasis, and the regulation of oxidative stress. Such studies in both AD and PD are essential for developing novel interventions that could mitigate the progression of these debilitating neurodegenerative diseases.

However, knowledge about the role of lactate and lactylation in other neurological disorders, such as HD, MS, MG, and EP, is limited. These conditions impose significant physiological and psychological burdens on patients, severely affecting their quality of life and even posing life-threatening risks. In particular, HD, an autosomal dominant neurodegenerative disease, is characterized by the progressive loss of neurons in the striatum and cerebral cortex, and patients with HD exhibit significant abnormalities in neuronal metabolism, disruptions in energy metabolic pathways, and mitochondrial dysfunction. However, research remains limited on the specific roles of lactate and lactylation in the pathophysiological processes of HD, as well as the interactions of related metabolic pathways and the regulatory mechanisms affecting neuronal survival and death.

This research imbalance between neurological disorders hinders the ability to comprehensively establish a network of associations between lactate, lactylation, and various disorder types. The lack of in-depth studies on diseases such as HD, MS, MG, and EP makes it challenging to identify potential common characteristics in lactate metabolism and lactylation across these seemingly distinct conditions. Furthermore, this gap in research impedes the current capacity to accurately analyze the unique pathogenesis and pathological processes of each disorder. As a result, the present understanding of the fundamental nature of these diseases is significantly restricted, hampering progress in developing both universal therapeutic strategies and personalized treatment plans based on lactate and lactylation.

## Summary and Future Prospects

Traditional research in the field of neuroscience has primarily focused on investigating the connections between neurotransmitters, ion channels, and genetic abnormalities in relation to neurological disorders. However, in recent years, there has been a notable emergence of studies linking lactate and lactylation to neurological diseases. This rapidly growing field of research is gradually identifying novel and significant perspectives on the pathological mechanisms and treatment of neurological disorders, injecting new energy into the established discipline.

Lactate, once considered merely a byproduct of anaerobic metabolism, is now gaining recognition in the field of neuroscience. Indeed, a growing body of research strongly suggests that lactate is a key mediator of brain metabolism. Furthermore, increasing evidence indicates that lactate is not only an important intermediate in brain energy metabolism but also a vital link between neurons and glial cells for energy transfer and metabolic regulation. From a metabolism perspective, when neuronal activity is heightened, glucose is extensively converted into lactate through glycolysis, and this lactate is then efficiently transported from astrocytes to neurons via specific transporters, such as MCTs, providing the stable and continuous energy supply essential for maintaining normal neuronal physiological functions. This process often occurs during critical physiological activities such as learning, memory consolidation, and neural signal transmission, highlighting the central role of lactate in the brain’s energy metabolism network. Furthermore, under specific physiological and pathological conditions, dynamic changes in lactate metabolism are closely associated with the functional state of the nervous system. Concurrently, lactylation, a newly recognized form of PTM, is gaining attention in neuroscience research. In particular, lactylation can profoundly impact protein structure, function, and intracellular localization without altering the genetic sequence. The role of this modification in the development and progression of neurological disorders has emerged as a key issue in neuroscience that requires urgent exploration. Indeed, in-depth studies of lactylation have the potential to uncover novel neural regulatory mechanisms, thus providing innovative approaches and methods for the diagnosis and treatment of neurological diseases.

The study of lactate and lactylation is of significant importance. Numerous studies have highlighted the relationship between the regulation of lactate metabolism and histone modification, indicating that protein lactylation plays a crucial role in various biological processes. However, most research has focused on diseases such as cancer, heart failure, and inflammation, while investigations into the impact of lactate and lactylation in the nervous system are still in their early stages, leaving many important questions unanswered. Therefore, a more comprehensive exploration of the role of lactate in physiological and pathological processes in the brain, along with the functional phenotypes and molecular mechanisms of protein lactylation, will enhance the current understanding of brain lactate metabolism and disease mechanisms.

However, there are several challenges in this field of research. First, significant variability exists in the research methods, sample types, and experimental conditions used across different neurological disorders. For instance, studies on AD often utilize brain tissue biopsy samples and cell models, while EP research tends to rely more on electroencephalogram monitoring and animal seizure models. This methodological variability complicates the identification of common patterns in findings, ultimately affecting the generalizability and accuracy of conclusions. Second, there is a lack of in-depth mechanistic studies in this area. Although reviews have addressed various diseases, research on the specific mechanisms of lactate and lactylation remains superficial for many conditions. For example, in relation to HD, while potential links between neuronal metabolic abnormalities and lactate metabolic pathways have been proposed, the precise molecular mechanisms and synergistic relationships with other pathogenic factors remain unclear. This gap in research makes it difficult to establish a comprehensive theoretical framework to guide clinical applications.

Moreover, neurological disorders are often chronic and progressive, necessitating long-term observation to ensure a comprehensive understanding of them. However, the existing studies are primarily cross-sectional or short-term longitudinal, meaning that they fail to capture the dynamic changes in lactate and lactylation over the extended course of disease development. This limitation hinders the assessment of the long-term usefulness of lactate and lactylation targets for disease prediction, diagnosis, and treatment. Although several studies have highlighted the role of lactate and lactylation in disease mechanisms, providing new therapeutic insights, there is a scarcity of research translating basic findings into clinical treatments. For example, in the case of MG, even though lactylation has been found to affect protein function at the neuromuscular junction, there is still a lack of in-depth exploration on how to develop effective therapeutic strategies (e.g., drug development and intervention) based on this finding. Therefore, there remains a significant gap between basic research and the real-world improvement of patient outcomes.

Nonetheless, despite certain limitations in the research, recent studies have increasingly suggested that lactate may emerge as a novel therapeutic target for neurological disorders. Diverging from previous research approaches, this review specifically focuses on the intrinsic connections between neurological diseases and lactate and lactylation. This work delves into cutting-edge research advancements in common neurological disorders such as AD and PD. In terms of AD research, this review not only outlines how disruptions in lactate metabolism affect the deposition and clearance of Aβ and the potential regulatory role of lactylation in the abnormal phosphorylation of tau proteinbut also examines the complex interactions between these mechanisms and both neuroinflammation and neuronal apoptosis. Regarding PD, this review emphasizes the impact of lactate level fluctuations on the survival and function of dopaminergic neurons, as well as the critical role of protein lactylation in regulating mitochondrial function and oxidative stress responses. As such, a novel perspective is provided on how understanding lactate metabolism and modification may be useful for elucidating the pathogenesis of these two common neurodegenerative diseases.

Notably, the novelty of this review lies in its extensive compilation of research related to relatively niche yet life-threatening neurological disorders such as HD, MS, MG, and EP. In the research on HD, although the exploration of the roles of lactate and lactylation is still in its early stages, the identified potential links between neuronal metabolic abnormalities and lactate metabolic pathways provide important clues for further in-depth studies. For MS, studies highlight the involvement of lactate in immune cell infiltration and myelin repair, thus revealing the unique role of lactate in the processes of autoimmune neural damage. In the context of MG, this review explores the impact of lactylation on protein function at the neuromuscular junction, offering new insights into the understanding of the neural signal transmission issues associated with the disease. Regarding EP, research is presented on the causal relationship between sharp changes in lactate levels during seizures and abnormal neuronal discharges, as well as on the role of lactylation in regulating epilepsy-related gene expression and neural plasticity. This comprehensive and systematic compilation of research significantly broadens the potential scope and depth of studies on lactate and lactylation in the field of neurological disorders and provides valuable theoretical foundations and research directions for uncovering common mechanisms across disease types and developing personalized therapeutic strategies.

In summary, exploring the specific molecular mechanisms of the role of lactate in neurological disorders and identifying precise therapeutic targets based on this represent a promising direction for future research in the neuroscience of neurological disorders. This line of inquiry not only offers novel theoretical explanations for the pathogenesis of neurological diseases but also establishes a solid foundation for developing efficient, safe, and targeted therapeutic strategies, providing new hope for patients affected by these conditions. Increasing evidence suggests that lactate plays a crucial role in brain metabolic processes, highlighting its potential as a therapeutic target. However, the specific molecular mechanisms of the role of lactate in neurological disorders and, thus, potential therapeutic targets remain to be fully elucidated. This gap in knowledge represents a significant opportunity for future research, which could ultimately enhance the current understanding of these complex diseases and lead to innovative treatment options.

## Data Availability

*Not applicable*.
